# Maintenance Therapy with Opium Tincture for Injecting Drug Users; Implications for Prevention from Viral Infections

**DOI:** 10.5812/hepatmon.8334

**Published:** 2013-04-16

**Authors:** Zahra Alam Mehrjerdi, Mehran Zarghami

**Affiliations:** 1 Iranian National Center for Addiction Studies (INCAS), Tehran University of Medical Sciences, Tehran, IR Iran; 2 Department of Psychiatry and Psychiatry and Behavioral Sciences Research Center, Mazandaran University of Medical Sciences, Sari, IR Iran

**Keywords:** Opium Tincture, Blood-Borne Pathogens, Health, Treatment

Dear Editor,

Viral infections such as HIV and HCV have a serious negative effect on the health of injecting drug users (IDUs) in Iran (1-4). Two recent studies in Tehran, Iran have shown that HCV prevalence was between 52%-80% among IDUs (5, 6). Furthermore, many IDUs might be infected with HIV and/or HCV in the earlier stages of their injection career, long before reaching therapeutic programs (7). As a result, simultaneous therapy of drug injecting and viral infections among IDUs is a medical priority. Opium tincture is a culturally acceptable alternative to medications such as methadone in some parts of south-east Asia and it is perceived as a traditional medicine for opioid detoxification and relieving opioid withdrawal symptoms. In some countries, however, the cost of methadone maintenance therapy is a barrier to its widespread use. One response to this problem has been the use of opium tincture as a less expensive substitution treatment. In recent years, maintenance therapy with opium tincture has been introduced in Iran as a new strategy to treat drug use problem among IDUs. In Myanmar, opium tincture has been known as an inexpensive pharmaceutical product which is as effective as methadone in treating opioid use problem (8). A study on opium-dependent patients has confirmed long-term positive therapeutic outcomes with opium tincture among opium-dependent patients (9). Somogyi and colleagues (2008) evaluated the clinical effectiveness of using different doses of opium tincture in the management of opioid withdrawal in a sample of 45 opium-dependent subjects and confirmed the effectiveness of opium tincture in the management of opioid withdrawal symptom with minimal adverse effects by using flexible dosing (10). A report by Natpratan (1996) showed that opium tincture was effective in treating opium dependence and contributed to managing drug abuse and HIV infection (8). In a study on a culturally-French environment that methadone was not available, buprenorphine and opium tincture were used in a group of 18 male and female opioid-dependent subjects during 14 months of treatment. The study results showed that body weight, physical and psychological health, socio-professional status and family relationships significantly improved in subjects after 14 months of using opium tincture (11). In Iran, opium use has a long history (12) but recently, seizures of heroin use have increased in Iran (13). Heroin seizures and heroin injection in Iran have contributed to making extreme efforts by policy makers and health providers to interrupt injection practices by widely implementing buprenorphine and methadone maintenance therapies (14-16). In 2010, the government of Iran also authorized the use of opium tincture as a main part of expanding maintenance therapies for opioid users especially IDUs. The expansion was approved after a pilot project was evaluated by the Iranian government (17). There is a strong body of evidence supporting buprenorphine and maintenance therapies for treating IDUs. As part of an international multicenter longitudinal cohort study with opioid maintenance therapy during 2003-2005, methadone maintenance therapy was administered to 127 opioid-dependent patients (65 were IDUs) in Tehran and the cases were followed for 6 months. A considerable reduction in using heroin and other opiates was reported by participants over the course of the study. Reductions occurred in the first three months of therapy and were maintained. Significant reductions in risky injecting were also observed among participants (18), but there is still a paucity of research on opium tincture as a long-term maintenance therapy for Iranian IDUs and its effectiveness in reducing the spread of viral infections such as HIV and HCV. This issue may be partly related to the new presence of opium tincture in the community. In general, drug use treatment is increasingly influenced by different types of drugs used. In Iran, using two forms of opioids are prevalent including opium and opium residues and a purified and potent form of heroin called heroin Kerack (not to be mistaken with Crack Cocaine) which has been cited as a potent motivation for injection practices (19). There is evidence that methadone maintenance therapy is not useful for some groups of drug users (20). Although opium tincture may be a suitable maintenance therapy for Iranian IDUs but it is important to explore its effectiveness in managing drug injection, and viral infections in comparison with highly approved methadone and buprenorphine maintenance therapies. Moreover, optimal dose amounts and frequencies for effective management of withdrawal symptoms must be investigated for IDUs. The primary objectives of maintenance therapies for IDUs include reduction and/or cessation of drug injection, risky behaviors such as shared injection practices and sharing injection equipment which could dramatically contribute to spreading viral infections. Such issues must be studied among Iranian IDUs who receive the maintenance therapy with opium tincture. The optimal duration of implementing the maintenance therapy with opium tincture and formal dose escalation for achieving positive treatment outcomes must be also evaluated. As a culturally and traditionally accepted medication, opium tincture can be an alternative pharmaceutical approach for treating IDUs in Iran but it is suggested to rigorously assess its effectiveness in managing drug-related signs and symptoms and viral infections among representative samples of IDUs before promoting a large-scale implementation in the country.
